# Urachal Sinus Presenting with Abscess Formation

**DOI:** 10.5402/2011/820924

**Published:** 2011-04-14

**Authors:** Jalal Eddine El Ammari, Youness Ahallal, Oussama El Yazami Adli, Mohammed Jamal El Fassi, My Hassan Farih

**Affiliations:** Department of Urology, University Hospital Center Hassan II Fes, Morocco

## Abstract

Urachal affections are rare. Their variable ways of presentation may represent a diagnostic challenge. Urachal sinuses are a rare type of these abnormalities. They are usually incidental findings and remain asymptomatic unless a complication (most commonly the infection) occurs. Infection of the urachal sinus would clinically present as purulent umbilical discharge, abdominal pain, and periumbilical mass. We report herein a case of infected urachal sinus in male adult. The diagnosis was suspected clinically and confirmed with ultrasonography and computed tomography scan. A preoperative cysto-fibroscopy showed normal aspect of the bladder and excluded sinus communication. An initial broad spectrum antibiotic therapy followed by complete excision of the sinus and fibrous tract without cuff of bladder has been therefore performed. The postoperative course was uneventful. No recurrence was observed after 18 months of followup. Histological examination did not reveal any sign of malignancy.

## 1. Introduction

Since the first description by Cabriolus in 1550, few cases of urachal sinuses have been reported in literature. Urachal abnormalities result from incomplete obliteration of the foetal urachus. They are rare in adults comparing to children [1]. Various types of remnants have been described and urachal sinus is the little common variety. The usual presenting symptom of this anomaly is umbilical discharge [2]. Diagnosis remains challenging due to the rarity of this lesion and the nonspecific nature of its symptomatology. This paper aims at reminding the diagnostic and therapeutic features of urachal sinus.

## 2. Case Report

**Figure 1:**
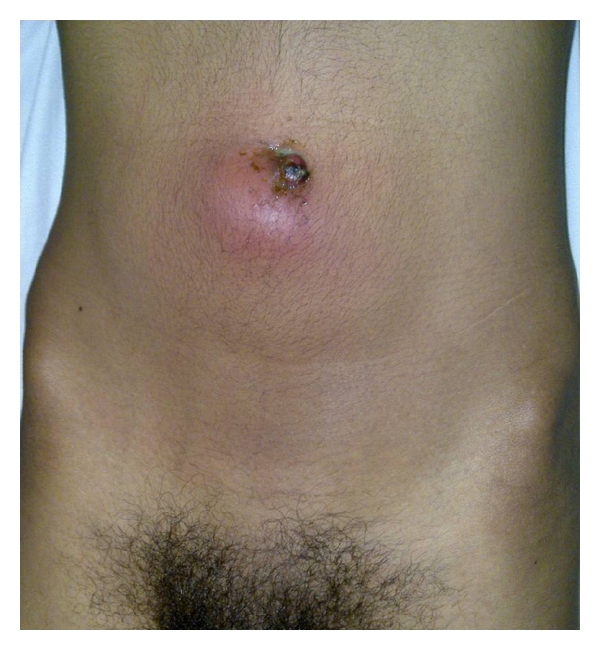
Purulent umbilical discharge with erythema and umbilical mass.

**Figure 2:**
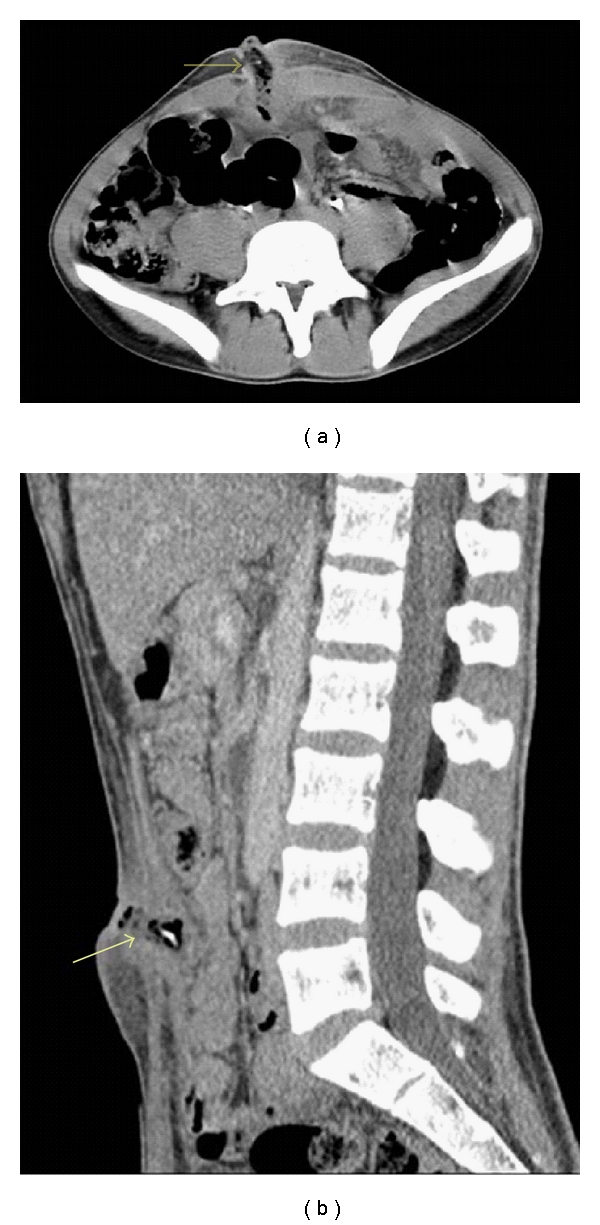
(a) and (b): heterogeneous urachal collection with calculus and gas formation communicating with the umbilicus.

A 22-year-old male patient, with no relevant past medical history, presented with a three days history of fever, abdominal pain, and umbilical discharge without digestive nor urinary symptoms. Physical examination revealed an initial temperature of 38.9°C, purulent umbilical discharge with erythema, and tender umbilical mass (Figure 1). Laboratory tests revealed marked leucocytosis of 24,000/mm^3^ and elevated C-reactive protein (42 mg/L). The urinalysis and renal function were within normal values. Culture of the umbilical discharge grew Klebsiella pneumonia, and blood culture was negative. Abdominal ultrasonography showed echoic collection in a midline cavity within the anterior abdominal wall. Computed tomography scan confirmed the diagnosis of infected urachal sinus showing a heterogeneous collection with calculus and gas formation communicating with the umbilicus (Figures 2(a) and 2(b)). 

**Figure 3:**
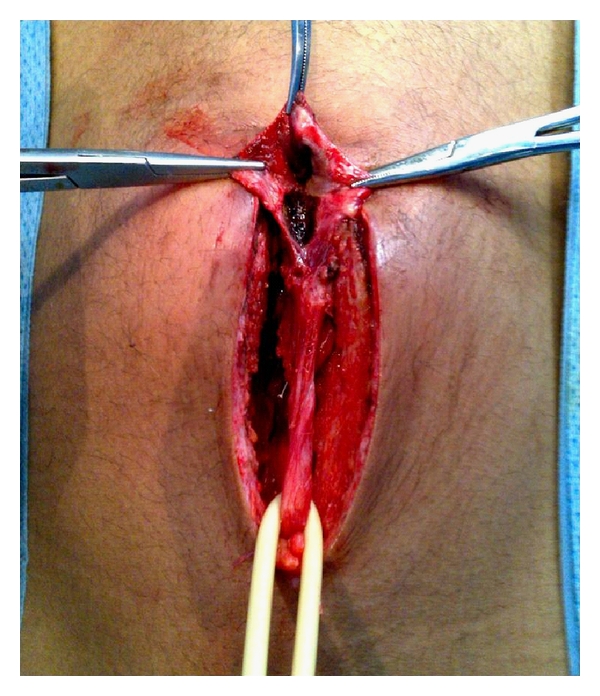
Infra-umbilical midline incision was used to excise the sinus and fibrous tract.

The patient was initially treated with intravenous antibiotics (ceftriaxone and gentamycine). Two days after apyrexia, cystoscopy and excision of the infected urachal sinus were performed simultaneously. Cystoscopy confirmed no evidence of a bladder anomaly. An infraumbilical midline incision was used to excise the sinus and fibrous tract (Figure 3). The postoperative course was uneventful. Histological examination did not reveal any signs of malignancy. No recurrence was observed after 18 months followup.

## 3. Discussion

The urachus is a vestigial remnant of at least two embryonic structures: the cloaca, and the allantois. The tubular urachus normally involutes before birth, remaining as a fibrous cord between the transversalis fascia anteriorly and the peritoneum posteriorly and attaches the umbilicus to the bladder dome.

Histologically, it presents with 3 layers: an innermost layer of modified transitional epithelium similar to the urothelium, a middle layer of fibro-connective tissue, and an outermost layer of smooth muscle continuing the detrusor [1, 3]. Usually presenting in early childhood, Urachal anomalies occur in a 2 : 1 male to female ratio with 2% ratio reported in adults [3]. 

Urachal abnormalities result from incomplete obliteration of the foetal urachus. There are five types of urachal abnormalities: (1) patent urachus, in which the entire tubular structure fails to close (50%); (2) urachal cyst, in which both ends of the canal close leaving an open central portion (30%); (3) urachal sinus, which drains proximally into the umbilicus (15%); (4) vesicourachal diverticulum, where the distal communication to the bladder persists (3–5%); and (5) alternating sinus, which can drain to either bladder or umbilicus [4, 5]. 

Urachal sinus abscess usually occurs by infection of mucinous secretion via the umbilicus. The commonly cultured microorganisms from the pus are Escherichia coli, Enterococcus faecium, Proteus, Streptococcus viridans and Fusobacterium [4, 5]. In our case, Klebsiella pneumonia was cultured.

The clinical signs and symptoms are nonspecific, as urachal sinus is largely asymptomatic until they become infected. However, the presence of the triad of symptoms including a tender midline infraumbilical mass, umbilical discharge and sepsis should arouse suspicion of urachal sinus [6].

Differential diagnosis of this condition includes anomalies of the vitelline ducts (such as Meckel's diverticulum), patent omphalomesenteric duct, infected umbilical vessel, appendicitis, or omphalitis [7].

Ultrasonography could help in establishing the diagnosis in 77% of patients. In our case, ultrasonography was not specific and computed tomography scan was used to confirm the diagnosis and analyse the connection to surrounding structures.

Urachal sinus can be complicated by stone and gaseous formation as was seen in our patient. Other reported complications include rupture into the peritoneal cavity leading to peritonitis, uracho-colonic fistula, and neoplastic transformation [4]. The risk of urachal malignancy in adults is high and the prognosis is poor [8]. 

Although the innermost layer of the urachus is mainly transitional cell, adenocarcinoma (mostly mucinous) is the predominant histological type. This is probably due to metaplasia arising from chronic inflammation. 

 Urachal cyst treatment depends on the presence of complications or associated conditions. Noninfected urachal sinus are usually removed in a single-step radical excision of the remnant which removes the entire lesion with or without a bladder cuff via open or laparoscopic surgical approach [9]. This intervention is performed to ovoid recurrence following simple drainage and to prevent developing malignant transformation [7]. In case of infection, a single-stage procedure backed with appropriate antibiotic therapy or 2-stage procedure involving initial incision and drainage, followed by later excision of the urachal remnant are adopted with uneventful postoperative course.

## 4. Conclusion

Infected urachal sinus is rare in adults. Presentation is atypical; therefore, a high index of suspicion is required in order to achieve a diagnosis. A triad of infraumbilical mass, umbilical discharge, and sepsis is suggestive. Ultrasound and computed tomography scan confirm the diagnosis and analyses the surrounding anatomical connections. An antibiotic regimen according to bacterial sensitivity is recommended prior to the surgical intervention. In order to prevent recurrence and malignant transformation, complete surgical excision with or without a bladder cuff is the standard treatment.
